# Supplementary material for: A low-cost and shielding-free ultra-low-field brain MRI scanner
Source: Nat Commun. 2021 Dec 14;12:7238. doi: 10.1038/s41467-021-27317-1 (PMC8671508; doi:10.1038/s41467-021-27317-1)
Supplement: Supplementary file 1 — Supplementary Information [file 41467_2021_27317_MOESM1_ESM.pdf]

Supplementary Information

A Low-cost and Shielding-free Ultra-low-field Brain MRI Scanner

Yilong Liu[1,2,a], Alex T. L. Leong[1,2,a], Yujiao Zhao[1,2,a], Linfang Xiao[1,2,a], Henry K. F. Mak[3], Anderson Tsang[4], Gary K. K. Lau[5], Gilberto K. K. Leung[4], Ed X. Wu[1,2,6*]

[1]Laboratory of Biomedical Imaging and Signal Processing, The University of Hong Kong, Pokfulam, Hong Kong SAR, China

[2]Department of Electrical and Electronic Engineering, The University of Hong Kong, Pokfulam, Hong Kong SAR, China

[3]Department of Diagnostic Radiology, LKS Faculty of Medicine, The University of Hong Kong, Pokfulam, Hong Kong SAR, China

[4]Department of Surgery, LKS Faculty of Medicine, The University of Hong Kong, Pokfulam, Hong Kong SAR, China

[5]Department of Medicine, LKS Faculty of Medicine, The University of Hong Kong, Pokfulam, Hong Kong SAR, China

[6]School of Biomedical Sciences, LKS Faculty of Medicine, The University of Hong Kong, Pokfulam, Hong Kong SAR, China

[a]Authors contributed equally to this work.

[*]Correspondence should be addressed to Ed X. Wu, Ph.D.:
Laboratory of Biomedical Imaging and Signal Processing, Department of Electrical and Electronic Engineering, School of Biomedical Sciences, The University of Hong Kong, Pokfulam, Hong Kong, Hong Kong SAR, China.
Fax: +852-2859-8738.
Tel: +852-3917-7096.
Email: ewu@eee.hku.hk

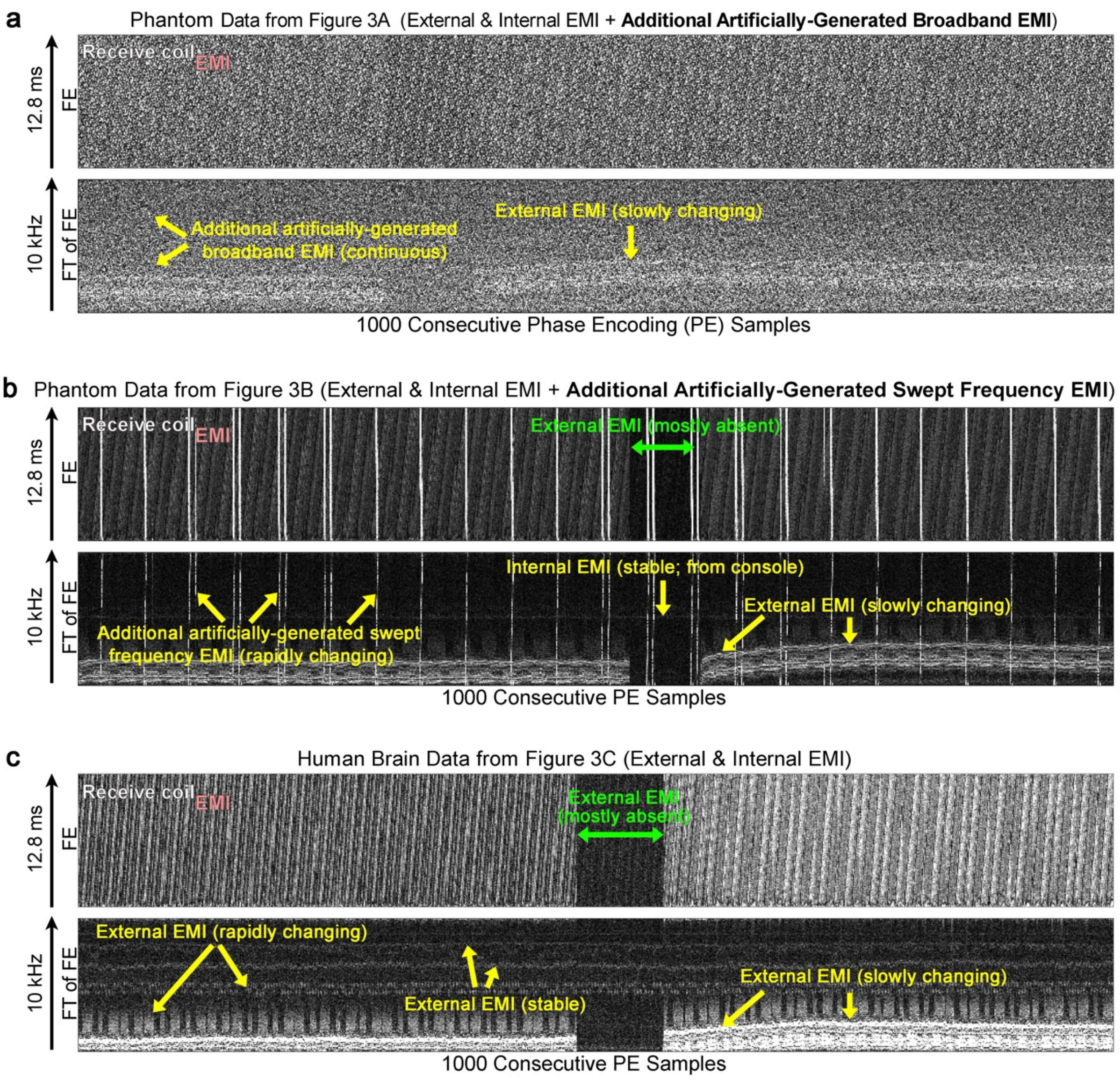

Supplementary Figure 1. Spectral analyses of the raw 3D fast spin echo (FSE) T2-weighted (T2W) data (corresponding to the spectral results shown in main Figure 3a-c, respectively, <u>without EMI elimination, and with RF transmit power off)</u> show the dynamically changing external and internal EMI sources. Note that, for illustration, only consecutive 1000 phase encoding (PE) samples, acquired over a ~72 s time span, were plotted along horizonal direction. Both frequency encoding (FE) line data and their Fourier transform magnitude (FT of FE) were shown. **a** Phantom data corresponding to main Figure 3a, with additional strong broadband EMI generated from a nearby source. **b** Phantom data corresponding to Figure 3b, with additional swept frequency EMI generated from a nearby

source (center frequency = 2.32 MHz, sweep span = 100 kHz, frequency points = 101, and continuous sweeping cycle period = 4 s). Note that narrowband EMI from an internal scanner source (console) was also visible. **c** Human brain data corresponding to Figure 3c, with signals from various external EMI sources. Spectral characteristics of these EMI sources during scan changed rapidly or gradually (indicated by yellow arrows) in terms of amplitude and frequency. Note that temporal changes of EMI characteristics are not obvious in the phantom data shown in (a) because the EMI artificially generated by the nearby broadband source overwhelmed the background EMI. Also note that, more external EMI sources can be identified in (c) partly because human body acted as an antenna and picked up more EMI signal. Nevertheless, the proposed EMI cancellation strategy worked robustly in presence of these temporally changing EMI signals from both narrowband and broadband sources, as supported by the spectral results in Figure 3.

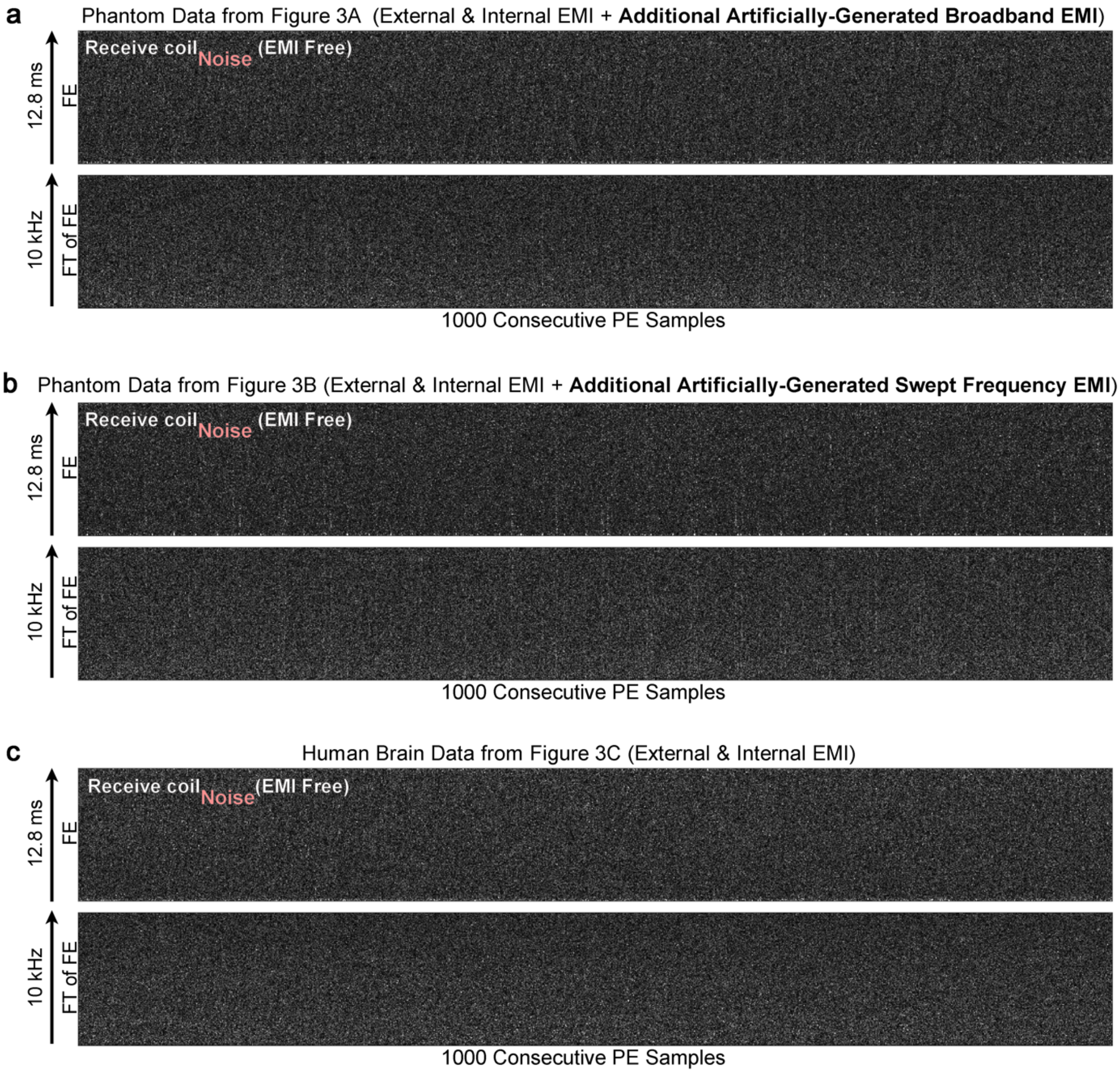

Supplementary Figure 2. Spectral analyses of the raw 3D FSE T2W data (corresponding to the magnitude spectral results shown in Figure 3a-c, respectively, with EMI elimination, and with RF transmit power off). All other descriptions were the same as those in Supplementary Fig. 1. By comparing with Supplementary Fig. 1, the proposed EMI cancellation strategy eliminated all EMI signals from various sources, as directly demonstrated by absence of any discernible EMI signals here. The results were displayed with enhanced brightness (×3) compared to Supplementary Fig. 1 for easy visualization here.

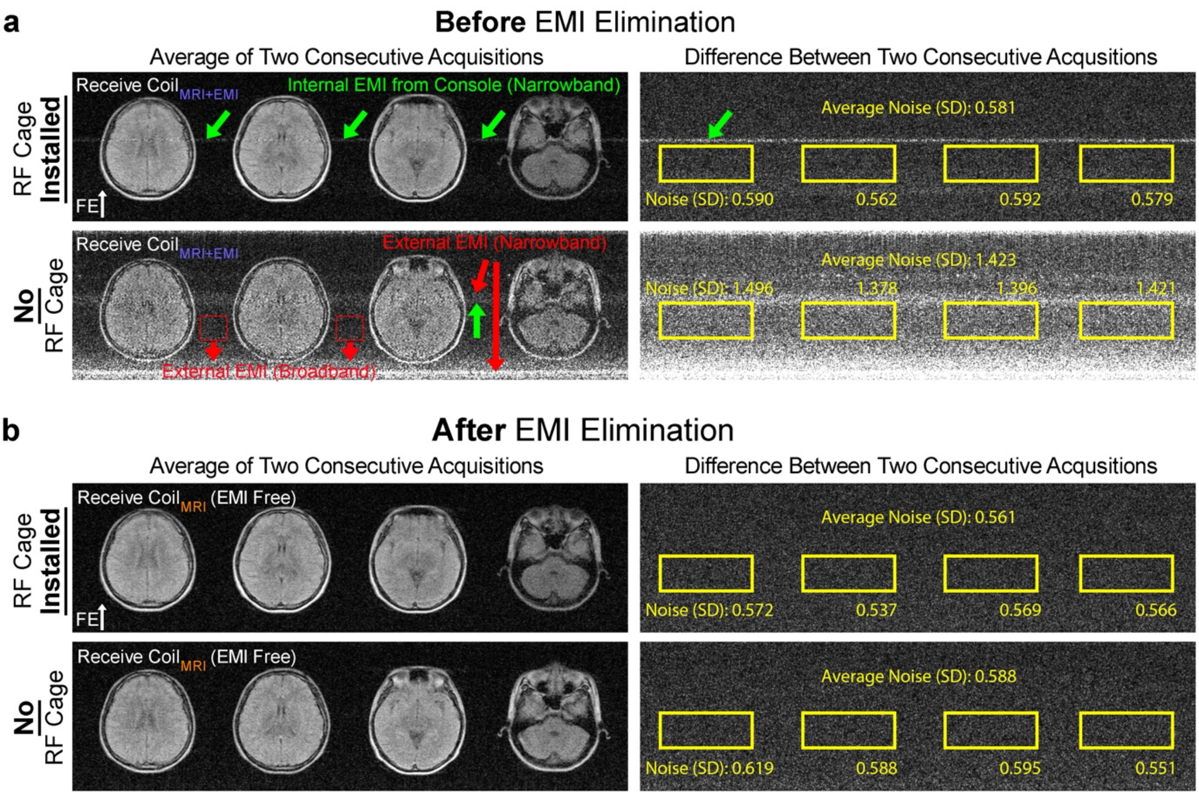

Supplementary Figure 3. In absence of RF shielding, our proposed deep learning driven EMI cancellation method achieves brain image noise levels as low as those obtained with the installation of a fully enclosed RF shielding cage (within 5% range). Representative 3D FSE fluid-attenuated inversion recovery (FLAIR) like images with number of excitations (NEX) = 2 (left; as average of two individual images), corresponding noise images (right; as the difference between two individual images), and noise level quantifications **a** before and **b** after EMI elimination. Two imaging experiments (with and without RF shielding cage) were performed in the same normal adult subject (23 yrs. old; male). FE direction was along the vertical direction. Note that, before EMI cancellation, a reduced and stable narrowband internal EMI signal was still present in the images when RF shielding cage was installed (due to MRI console EMI leakage, as indicated by green arrows). Without RF shielding cage, both narrowband and wideband external EMI signals were present (indicated by red arrows). The image noise levels were quantified here from the difference image between the first and second individual complex images[1]. Without deep learning EMI elimination procedure, the average

noise level without RF shielding cage was significantly higher (1.423 in standard deviation or SD) than that with RF shielding cage (0.581) as expected. After deep learning EMI elimination, the average noise level without RF shielding cage significantly decreased to 0.588 (from 1.423), which was only 1.2% higher than that obtained with RF shielding cage before EMI elimination (0.581) and 4.8% higher than that after EMI elimination (0.561). The EMI elimination procedure provided nearly complete removal of EMI noise in the images. That is, in absence of EMI shielding cage, it led to final image noise levels as low as those obtained with a fully enclosed RF shielding cage installed (within 5% range) in human brain imaging experiments. No conductive shielding cloth or EMI pickup electrodes were used in this study.

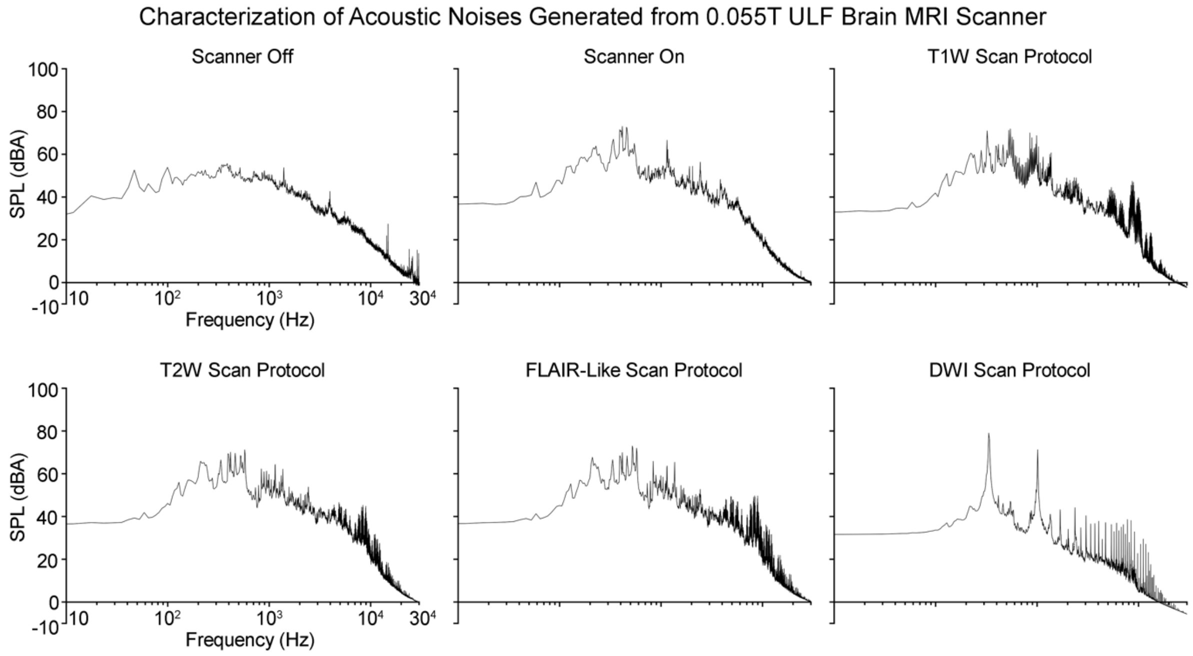

Supplementary Figure 4. Acoustically quiet MRI scanning at 0.055 T. A-weighted filtered power spectra of the respective measured background noise when the scanner was off (top left), and acoustic noise generated when the scanner was on (top middle) and during the running of T1-weighted (T1W) (top right), T2W (bottom left), FLAIR (bottom middle) and diffusion-weighted imaging (DWI) (bottom right) protocols. The microphone was placed isocenter in the middle of the MRI receive coil. The maximum peak sound pressure level (SPL) during scanning was generally low (i.e., <85 dBA during DWI scanning).

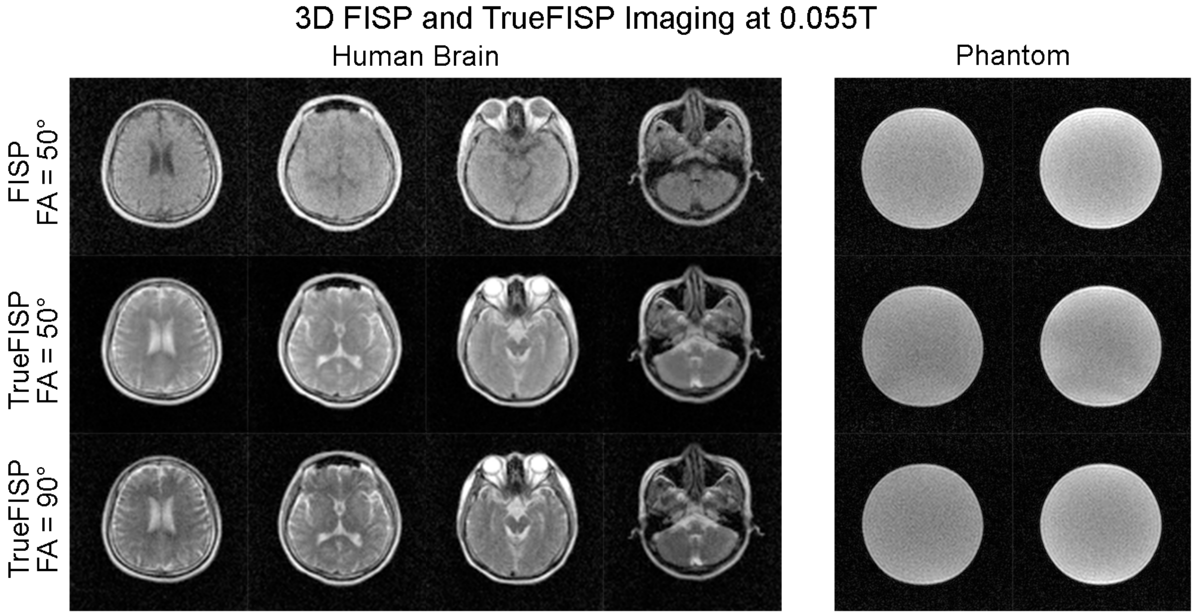

Supplementary Figure 5. Preliminary 3D fast imaging with steady-state free precession (FISP) and true FISP (TrueFISP) results at 0.055 T. Representative axial sections (32 yrs. old; male) were acquired with sequence parameters TR/TE = 7.4/3.7 ms, 96x96x16 acquisition matrix, and scan time 9 mins for TrueFISP; and TR/TE=20/7 ms, and scan time 9 mins for FISP. Slice thickness was 10 mm.

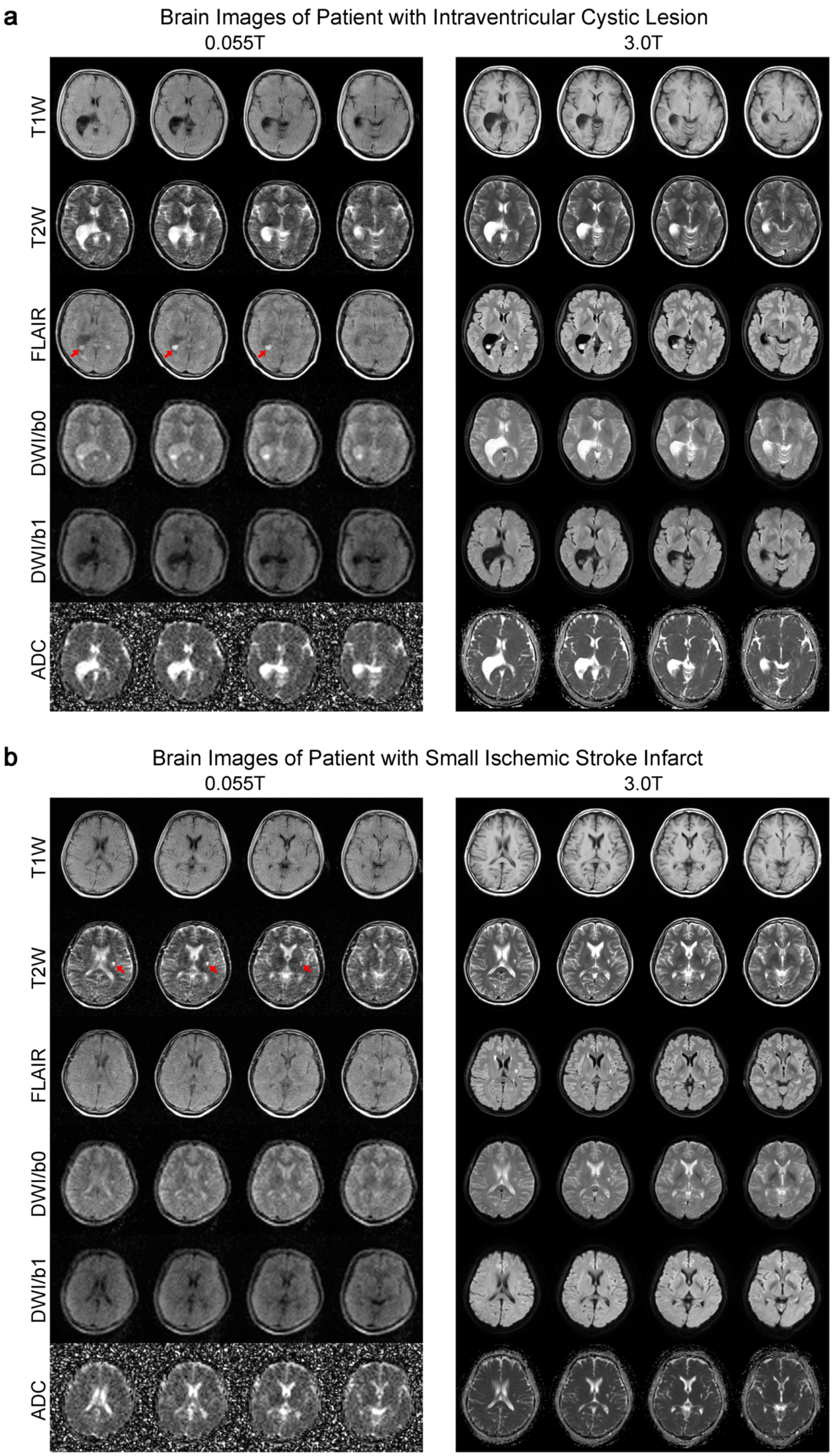
a
Brain Images of Patient with Intraventricular Cystic Lesion
0.055T
3.0T
T1W
T2W
FLAIR
DWI/b0
DWI/b1
ADC
b
Brain Images of Patient with Small Ischemic Stroke Infarct
0.055T
3.0T
T1W
T2W
FLAIR
DWI/b0
DWI/b1
ADC

Supplementary Figure 6. Clinical utility of 0.055 T MRI for examining rare lesions and small ischemic stroke infarcts. a Patient (45 yrs. old; female) with intraventricular cystic lesion. T1 images showed clear asymmetry between the right and left hemisphere, especially in the right lateral ventricle, indicating the presence of a lesion. Choroid plexus cyst was visible particularly at the occipital horn of the right lateral ventricle in 0.055 T FLAIR images, which showed excellent correspondence with 3 T results (indicated by red arrows). **b** Chronic (>3 weeks) ischemic stroke patient (57 yrs. old; female). Small lacunar infarcts (~3x3 mm^2; indicated by red arrows) were visible at left putamen in 0.055 T and 3 T images. Total scan time was about 30 mins at 0.055 T for each patient. Patients were also scanned by a clinical 3 T MRI scanner using the standard clinical protocols (~20 mins total scan time) on the same day.

Reference

1. Xie, V.B., Lyu, M. & Wu, E.X. EPI Nyquist ghost and geometric distortion correction by two-frame phase labeling. *Magn Reson Med* **77**, 1749-1761 (2017).
