# Supplementary material for: A low-cost and shielding-free ultra-low-field brain MRI scanner
Source: Nat Commun. 2021 Dec 14;12:7238. doi: 10.1038/s41467-021-27317-1 (PMC8671508; doi:10.1038/s41467-021-27317-1)
Supplement: Supplementary file 3 — Reporting Summary [file 41467_2021_27317_MOESM3_ESM.pdf]

nature portfolio

Corresponding author(s): Ed X. WU

Last updated by author(s): Oct 26, 2021

Reporting Summary

Nature Portfolio wishes to improve the reproducibility of the work that we publish. This form provides structure for consistency and transparency in reporting. For further information on Nature Portfolio policies, see our Editorial Policies and the Editorial Policy Checklist.

Statistics

For all statistical analyses, confirm that the following items are present in the figure legend, table legend, main text, or Methods section.

n/a	Confirmed	
☐	☒	The exact sample size (*n*) for each experimental group/condition, given as a discrete number and unit of measurement
☐	☒	A statement on whether measurements were taken from distinct samples or whether the same sample was measured repeatedly
☒	☐	The statistical test(s) used AND whether they are one- or two-sided<br>*Only common tests should be described solely by name; describe more complex techniques in the Methods section.*
☒	☐	A description of all covariates tested
☒	☐	A description of any assumptions or corrections, such as tests of normality and adjustment for multiple comparisons
☐	☒	A full description of the statistical parameters including central tendency (e.g. means) or other basic estimates (e.g. regression coefficient) AND variation (e.g. standard deviation) or associated estimates of uncertainty (e.g. confidence intervals)
☒	☐	For null hypothesis testing, the test statistic (e.g. *F*, *t*, *r*) with confidence intervals, effect sizes, degrees of freedom and *P* value noted<br>*Give P values as exact values whenever suitable.*
☒	☐	For Bayesian analysis, information on the choice of priors and Markov chain Monte Carlo settings
☒	☐	For hierarchical and complex designs, identification of the appropriate level for tests and full reporting of outcomes
☒	☐	Estimates of effect sizes (e.g. Cohen's *d*, Pearson's *r*), indicating how they were calculated

Our web collection on statistics for biologists contains articles on many of the points above.

Software and code

Policy information about availability of computer code

Data collection	MRI data collection was performed with Powerscan™ software v6.3 provided with the MR Solutions EVO console (Guildford, UK).
Data analysis	MRI data were analyzed with custom codes in MATLAB 2018b and PyTorch 1.8.1 package, and can be downloaded from a public repository (https://github.com/bispmri/Ultra-low-field-MRI-Scanner).

For manuscripts utilizing custom algorithms or software that are central to the research but not yet described in published literature, software must be made available to editors and reviewers. We strongly encourage code deposition in a community repository (e.g. GitHub). See the Nature Portfolio guidelines for submitting code & software for further information.

Data

Policy information about availability of data

All manuscripts must include a data availability statement. This statement should provide the following information, where applicable:

- Accession codes, unique identifiers, or web links for publicly available datasets
- A description of any restrictions on data availability
- For clinical datasets or third party data, please ensure that the statement adheres to our policy

All main data used, analyzed and generated that support the findings of this study, and key technical documents are available for download from a public repository (https://github.com/bispmri/Ultra-low-field-MRI-Scanner). The data points generated for the magnitude averaged spectra of raw MRI FE lines in Fig. 3 and the A-weighted filtered power spectra of sound pressure level recordings in Supplementary Fig. 4 are provided with this paper as a source data file. Other information is available from the corresponding author upon reasonable request.

Field-specific reporting

Please select the one below that is the best fit for your research. If you are not sure, read the appropriate sections before making your selection.

☒ Life sciences ☐ Behavioural & social sciences ☐ Ecological, evolutionary & environmental sciences

For a reference copy of the document with all sections, see nature.com/documents/nr-reporting-summary-flat.pdf

Life sciences study design

All studies must disclose on these points even when the disclosure is negative.

Sample size	Phantom experiments were first conducted to optimize image quality when imaging in radiofrequency (RF) and magnetic shielding-free environments. Human brain imaging experiments were then conducted on healthy volunteers before patient groups. Sample size for human brain imaging experiments were determined with consultation of a senior neuroradiologist who examined the quality of the MRI images, while being blinded to the corresponding clinical 3 Tesla MRI images.
Data exclusions	Some human brain imaging data from patient groups were excluded either due to chest access issues at the 0.055 Tesla MRI scanner or due to deteriorating medical condition before scheduled scans at 0.055 Tesla or/and clinical 3 Tesla MRI.
Replication	Imaging results were highly replicable in phantoms and human subjects. Human brain imaging experiments in particular were performed more than 100 times in healthy subjects.
Randomization	Randomization was not applicable, as the goal of the study was qualitative image validation of human brain images acquired with patient groups at ultra-low-field (ULF, 0.055 Tesla) MRI and comparison with those acquired at clinical high-field (3 Tesla) MRI scanners.
Blinding	One senior radiologist read and evaluated the brain images acquired with patient groups. The 0.055 Tesla images were read and evaluated first while blinded to the corresponding 3 T images.

Reporting for specific materials, systems and methods

We require information from authors about some types of materials, experimental systems and methods used in many studies. Here, indicate whether each material, system or method listed is relevant to your study. If you are not sure if a list item applies to your research, read the appropriate section before selecting a response.

Materials & experimental systems

n/a	Involved in the study	
☒	☐	Antibodies
☒	☐	Eukaryotic cell lines
☒	☐	Palaeontology and archaeology
☒	☐	Animals and other organisms
☐	☒	Human research participants
☒	☐	Clinical data
☒	☐	Dual use research of concern

Methods

n/a	Involved in the study	
☒	☐	ChIP-seq
☒	☐	Flow cytometry
☐	☒	MRI-based neuroimaging

Human research participants

Policy information about studies involving human research participants

Population characteristics	The MRI subjects were healthy volunteers and patient groups. Healthy volunteers consist of male and female subjects of age 18-70. Patient groups consist of male and female subjects of age 30-80, who are presently clinically diagnosed with subacute/chronic stroke, subacute/chronic intracerebral hemorrhage, and benign tumors (e.g., meningioma).
Recruitment	Healthy subjects were recruited via informational fliers and e-mails through The University of Hong Kong. The patients were recruited by investigators at the The University of Hong Kong, specifically the neurology and neurosurgery clinics at Queen Mary Hospital, Hong Kong. There were no no self-selection biases or other biases throughout the recruitment process.
Ethics oversight	The study protocol was approved by The University of Hong Kong/Hospital Authority Hong Kong West Cluster (HKU/HA HKW IRB), and written informed consent was obtained prior to any MRI examination.

Note that full information on the approval of the study protocol must also be provided in the manuscript.

Magnetic resonance imaging

Experimental design

Design type	Structural neuroimaging.
Design specifications	T1-weighted (T1W), T2-weighted (T2W), fluid-attenuated inversion recovery (FLAIR), and diffusion-weighted imaging (DWI) images were acquired in phantoms and human subjects (both healthy subjects and patient groups). No performance/task-based/resting-state functional MRI or clinical trials were performed.
Behavioral performance measures	No behavioral tasks were performed.

Acquisition

Imaging type(s)	Structural and diffusion-weighted neuroimaging.
Field strength	0.055 Tesla and 3 Tesla.
Sequence & imaging parameters	0.055 Tesla: T1W and T2W scan protocols were acquired with 3D gradient-echo (GRE; TR/TE = 52/13 ms, FA = 40°, acquisition matrix = 128x128x32, FOV = 250x250x320 mm3, acquisition slice thickness = 10 mm, and NEX = 2) and 3D fast spin echo (FSE; TR/TE = 1500/202 ms, FA = 90/180°, ETL = 21, acquisition matrix = 128x126x32, FOV = 250x250x320 mm3, acquisition slice thickness = 10 mm, and NEX = 2). FLAIR scan protocol parameters were similar to those for T2W protocol above with FSE parameters TR/TE = 500/129 ms, ETL = 13, acquisition matrix = 128x117x32, NEX = 4, and scan time 7.5 mins. DWI scan protocol was implemented with a 2D spin-echo EPI using a pair of diffusion gradients. The parameters were TR/TE = 2800/102 ms, acquisition matrix = 64x64, FOV = 250x250 mm2, acquisition slice thickness/slice gap = 10/0 mm, diffusion time/duration Δ/δ = 49/30 ms, NEX = 52 for DWI images with b = 0 (i.e., b0 images) and images with b = 500 s/mm2 (i.e., b1 images) diffusion weighting along three orthogonal directions. Apparent diffusion coefficient (ADC) maps were calculated from b0 and b1 images. All image reconstruction procedures above were based on Fourier transform of fully sampled data. All images were reconstructed to 1x1x5 mm3 display resolution by applying zero padding in k-space.<br><br>3 Tesla: T1W and T2W images were acquired with 2D FSE (T1W: TR/TE = 2700/25 ms, inversion time = 830 ms, FA = 111°, ETL = 8, acquisition matrix = 340x280, FOV = 230x230 mm2, acquisition slice thickness/slice gap = 5/0.5 mm, 27 slices, and NEX = 1; T2W: TR/TE = 5900/106 ms, FA = 120°, ETL = 30, acquisition matrix = 448x448, FOV = 230x230 mm2, acquisition slice thickness/slice gap = 5/0.5 mm, 27 slices, and NEX = 2). FLAIR images were acquired with 3D FSE with TR/TE = 6300/104 ms, inversion time = 1800 ms, FA = 90/180°, ETL = 180, acquisition matrix = 256x256x60, FOV = 250x250x150 mm3, acquisition slice thickness = 2.5 mm, and NEX = 1. DWI images were acquired with a 2D spin-echo EPI using a diffusion gradient pair. The parameters were TR/TE = 4000/57 ms, acquisition matrix = 120x160, FOV = 230x230 mm2, acquisition slice thickness = 5 mm, 54 slices, NEX = 4 for images with b = 0 (i.e., b0 images) and images with b = 1000 s/mm2 (i.e., b1 images) diffusion weighting along three orthogonal directions. The acquisition resolution was 0.7x0.8x5.0 mm3, 0.5x0.5x5.0 mm3, 1.0x1.0x4.0 mm3, and 1.9x1.4x5.0 mm3, respectively.
Area of acquisition	Brain.

Diffusion MRI ☒ Used ☐ Not used

Parameters	0.055 Tesla: b = 0 (i.e., b0 images) and images with b = 500 s/mm2 (i.e., b1 images) diffusion weighting along three orthogonal directions with NEX = 52.<br><br>3 Tesla: b = 0 (i.e., b0 images) and images with b = 1000 s/mm2 (i.e., b1 images) diffusion weighting along three orthogonal directions with NEX = 4.

Preprocessing

Preprocessing software	MATLAB 2018b was used to process the imaging data.
Normalization	No normalization procedure was used.
Normalization template	No normalization template was used.
Noise and artifact removal	For T1, T2 and FLAIR, images were denoised with BM4D. No artifact removal procedures were performed. For diffusion-weighted imaging (DWI), both Nyquist ghosts and field inhomogeneity related geometric distortions were corrected when reconstructing b0 and b1 images. This was implemented with custom MATLAB image-reconstruction code.
Volume censoring	No custom image masks were used for any image processing steps.

Statistical modeling & inference

Model type and settings	No statistical modeling and inference were used.
Effect(s) tested	No tasks or stimulus effects were tested.

Specify type of analysis: ☐ Whole brain ☐ ROI-based ☐ Both

Statistic type for inference (See Eklund et al. 2016)	No statistical modeling and inference were used.
Correction	No statistical modeling and inference were used.

Models & analysis

n/a	Involved in the study
☒	☐ Functional and/or effective connectivity
☒	☐ Graph analysis
☒	☐ Multivariate modeling or predictive analysis
